# Organ manifestations of COVID-19: what have we learned so far (not only) from autopsies?

**DOI:** 10.1007/s00428-022-03319-2

**Published:** 2022-04-01

**Authors:** Danny Jonigk, Christopher Werlein, Till Acker, Martin Aepfelbacher, Kerstin U. Amann, Gustavo Baretton, Peter Barth, Rainer M. Bohle, Andreas Büttner, Reinhard Büttner, Reinhard Dettmeyer, Philip Eichhorn, Sefer Elezkurtaj, Irene Esposito, Katja Evert, Matthias Evert, Falko Fend, Nikolaus Gaßler, Stefan Gattenlöhner, Markus Glatzel, Heike Göbel, Elise Gradhand, Torsten Hansen, Arndt Hartmann, Axel Heinemann, Frank L. Heppner, Julia Hilsenbeck, David Horst, Jan C. Kamp, Gita Mall, Bruno Märkl, Benjamin Ondruschka, Jessica Pablik, Susanne Pfefferle, Alexander Quaas, Helena Radbruch, Christoph Röcken, Andreas Rosenwald, Wilfried Roth, Martina Rudelius, Peter Schirmacher, Julia Slotta-Huspenina, Kevin Smith, Linna Sommer, Konrad Stock, Philipp Ströbel, Stephanie Strobl, Ulf Titze, Gregor Weirich, Joachim Weis, Martin Werner, Claudia Wickenhauser, Thorsten Wiech, Peter Wild, Tobias Welte, Saskia von Stillfried, Peter Boor

**Affiliations:** 1 Institute of Pathology, Hannover Medical School, Hannover, Germany; 2 Institute of Neuropathology, University Hospital Giessen and Marburg, Giessen, Germany; 3 Institute of Medical Microbiology, Virology, and Hygiene, University Medical Center Hamburg-Eppendorf, Hamburg, Germany; 4 Department of Nephropathology, University Hospital Erlangen-Nürnberg, Erlangen, Germany; 5 Department of Pathology, University Hospital Dresden, Dresden, Germany; 6 Gerhard Domagk Institute of Pathology, University Hospital Münster, Münster, Germany; 7 Department of Pathology, University Hospital Saarland Homburg, Homburg, Germany; 8 Institute of Forensic Medicine, University Medical Center Rostock, Rostock, Germany; 9 Department of Pathology, University Hospital Cologne, Cologne, Germany; 10 Department of Legal Medicine, University Hospital Giessen and Marburg, Giessen, Germany; 11 Department of Pathology, University Hospital Erlangen-Nürnberg, Erlangen, Germany; 12 Department of Pathology, Charité - Universitätsmedizin Berlin, corporate member of Freie Universität Berlin and Humboldt-Universität zu Berlin, Berlin, Germany; 13 Department of Pathology, University Hospital Düsseldorf, Düsseldorf, Germany; 14 Department of Pathology, University Hospital Regensburg, Regensburg, Germany; 15 Department of Pathology, University Hospital Tübingen, Tübingen, Germany; 16 Department of Pathology, University Hospital Jena, Jena, Germany; 17 Department of Pathology, University Hospital Giessen and Marburg, Giessen, Germany; 18 Institute of Neuropathology, University Medical Center Hamburg-Eppendorf, Hamburg, Germany; 19 Senckenberg Institute of Pathology, University Hospital Frankfurt, Frankfurt, Germany; 20 Department of Pathology, University Hospital OWL of the Bielefeld University, Campus Lippe, Detmold, Germany; 21 Department of Legal Medicine, University Medical Center Hamburg-Eppendorf, Hamburg, Germany; 22 Department of Neuropathology, Charité - Universitätsmedizin Berlin, corporate member of Freie Universität Berlin and Humboldt-Universität zu Berlin, Berlin, Germany; 23 German Center for Neurodegenerative Diseases (DZNE) Berlin, Berlin, Germany; 24 Cluster of Excellence, NeuroCure, Berlin, Germany; 25 Department of Respiratory Medicine, Hannover Medical School, Hannover, Germany; 26 Department of Legal Medicine, University Hospital Jena, Jena, Germany; 27 General Pathology and Molecular Diagnostics, University Hospital Augsburg, Augsburg, Germany; 28 Institute of Legal Medicine, University Medical Center Hamburg-Eppendorf, Hamburg, Germany; 29 Department of Pathology, University Hospital Schleswig-Holstein, Kiel, Germany; 30 Institute of Pathology, University of Würzburg, Würzburg, Germany; 31 Department of Pathology, University Medical Center Mainz, Mainz, Germany; 32 Institute of Pathology, Ludwig-Maximilians-Universität Munich, Munich, Germany; 33 Department of Pathology, Heidelberg University Hospital, Heidelberg, Germany; 34 Department of Pathology, TUM School of Medicine of Technical University of Munich, Munich, Germany; 35 Department of Nephrology, TUM School of Medicine of Technical University of Munich, Munich, Germany; 36 Department of Pathology, University Medical Center Göttingen, Göttingen, Germany; 37 Department of Neuropathology, University Hospital RWTH Aachen, Aachen, Germany; 38 Institute for Surgical Pathology, Medical Center, University of Freiburg, Freiburg, Germany; 39 Department of Pathology, University Hospital Halle (Saale), Halle (Saale), Germany; 40 Department of Pathology, University Medical Center Hamburg-Eppendorf, Hamburg, Germany; 41 Institute of Pathology, University Hospital RWTH Aachen, Aachen, Germany; 42 Department of Nephrology and Immunology, University Hospital RWTH Aachen, Aachen, Germany

**Keywords:** SARS-CoV-2, Diffuse alveolar damage, Acute kidney damage, Immune response

## Abstract

The use of autopsies in medicine has been declining. The COVID-19 pandemic has documented and rejuvenated the importance of autopsies as a tool of modern medicine. In this review, we discuss the various autopsy techniques, the applicability of modern analytical methods to understand the pathophysiology of COVID-19, the major pathological organ findings, limitations or current studies, and open questions. This article summarizes published literature and the consented experience of the nationwide network of clinical, neuro-, and forensic pathologists from 27 German autopsy centers with more than 1200 COVID-19 autopsies. The autopsy tissues revealed that SARS-CoV-2 can be found in virtually all human organs and tissues, and the majority of cells. Autopsies have revealed the organ and tissue tropism of SARS-CoV-2, and the morphological features of COVID-19. This is characterized by diffuse alveolar damage, combined with angiocentric disease, which in turn is characterized by endothelial dysfunction, vascular inflammation, (micro-) thrombosis, vasoconstriction, and intussusceptive angiogenesis. These findings explained the increased pulmonary resistance in COVID-19 and supported the recommendations for antithrombotic treatment in COVID-19. In contrast, in extra-respiratory organs, pathological changes are often nonspecific and unclear to which extent these changes are due to direct infection vs. indirect/secondary mechanisms of organ injury, or a combination thereof. Ongoing research using autopsies aims at answering questions on disease mechanisms, e.g., focusing on variants of concern, and future challenges, such as post-COVID conditions. Autopsies are an invaluable tool in medicine and national and international interdisciplinary collaborative autopsy-based research initiatives are essential.

**Supplementary Information:**

The online version contains supplementary material available at 10.1007/s00428-022-03319-2.

## Introduction

The COVID-19 pandemic has clearly highlighted the high importance of autopsies as an integral part of modern medicine. COVID-19 autopsies have provided insights into cellular and molecular pathomechanisms [8] by allowing direct analyses of the affected organs, thereby providing profound and viable data supporting concepts for therapeutic approaches, e.g., the striking absence of lymphocytic myocarditis despite suggestive clinical presentation [59]. Autopsies also documented the role of pre-existing medical conditions and vulnerable patients, provided the ultimate causes of death, and provided feedback on the effectiveness of treatment strategies [43, 106]. At the beginning of this global health crisis, post-mortem examinations revealed important histopathological findings on the nature of SARS-CoV-2 infection, such as the pronounced thrombotic angiopathy in the lungs [162] and the high incidence of thromboembolic events [157]. Despite this, some countries still discourage, or have even forbidden, COVID-19 autopsies [66, 133].

To facilitate the systematic evaluation of autopsy findings, research endeavors and multicentric trials, surgical pathologists, neuropathologists, and forensic pathologists, supported by virologists, scientific, and professional societies and healthcare authorities, combined forces to build the German Research Network for Autopsies in Pandemics (DEFEAT PANDEMIcs). DEFEAT PANDEMIcs established a highly organized nationwide network to collect and share data, materials, and findings as swiftly as possible. This has strengthened the comprehensive medical research of COVID-19, with over 90 papers published by the parties involved so far. DEFEAT PANDEMIcs also serves as a foundation for potential future pandemics, by providing standard operating procedures and emergency plans for autopsies, validating innovative techniques for sample collection and tissue analysis, and creating structures for systematic reporting in outbreak scenarios. Within this framework, a key building block is the nationwide registry of COVID-19 autopsies (DeRegCOVID; www.DeRegCOVID.ukaachen.de), which has been operational since early April 2020. The DeRegCOVID provides an electronic backbone for centralized and coordinated support for autopsy centers and researchers, for data reporting, biomaterial availability, and structured data coordination [148, 151] with the benefit of decentralized sample storage.

This article summarizes autopsy techniques and major COVID-19 associated pathological organ findings, based on literature review and a synoptic contemplation from over 1200 autopsies within DeRegCOVID performed and analyzed by the DEFEAT PANDEMIcs centers (for details, see Supplementary Table 1). Each specific organ pathology section was primarily prepared by national reference centers for the particular organ and consented by the consortium.

## Autopsy types and techniques

Autopsies of COVID-19 patients, and autopsies in general, can be divided into (a) clinical, (b) ordered by health authorities, or (c) forensic autopsies. A clinical autopsy is most often initiated by clinicians rather than the relatives of the deceased and performed in cases with assumed natural causes of death. Consent is required either from the patient himself (generally in the case of a research project), or from the next of kin. These autopsies are traditionally performed by surgical pathologists, and in the majority of cases, detailed clinical history and data are available; health care authorities can also order autopsies. In Germany, this is done following the Infection Protection Law (Infektionsschutzgesetz) and does not require patient or relative consent; however, this is obtained where possible. These autopsies are performed by both surgical and forensic pathologists [43]. If death occurs at home, clinical data is generally not available prior to autopsy. However, such autopsies provide important insights into causes of death of non-hospitalized patients, and can help to uncover previously unrecognized pathologies, such as asymptomatic infections [83]. Finally, a forensic autopsy is always performed by forensic pathologists and is carried out in cases of (suspected or proven) unnatural causes of death, such as trauma, suicide, homicide, or when the cause of death is unclear.

**Fig. 1:**
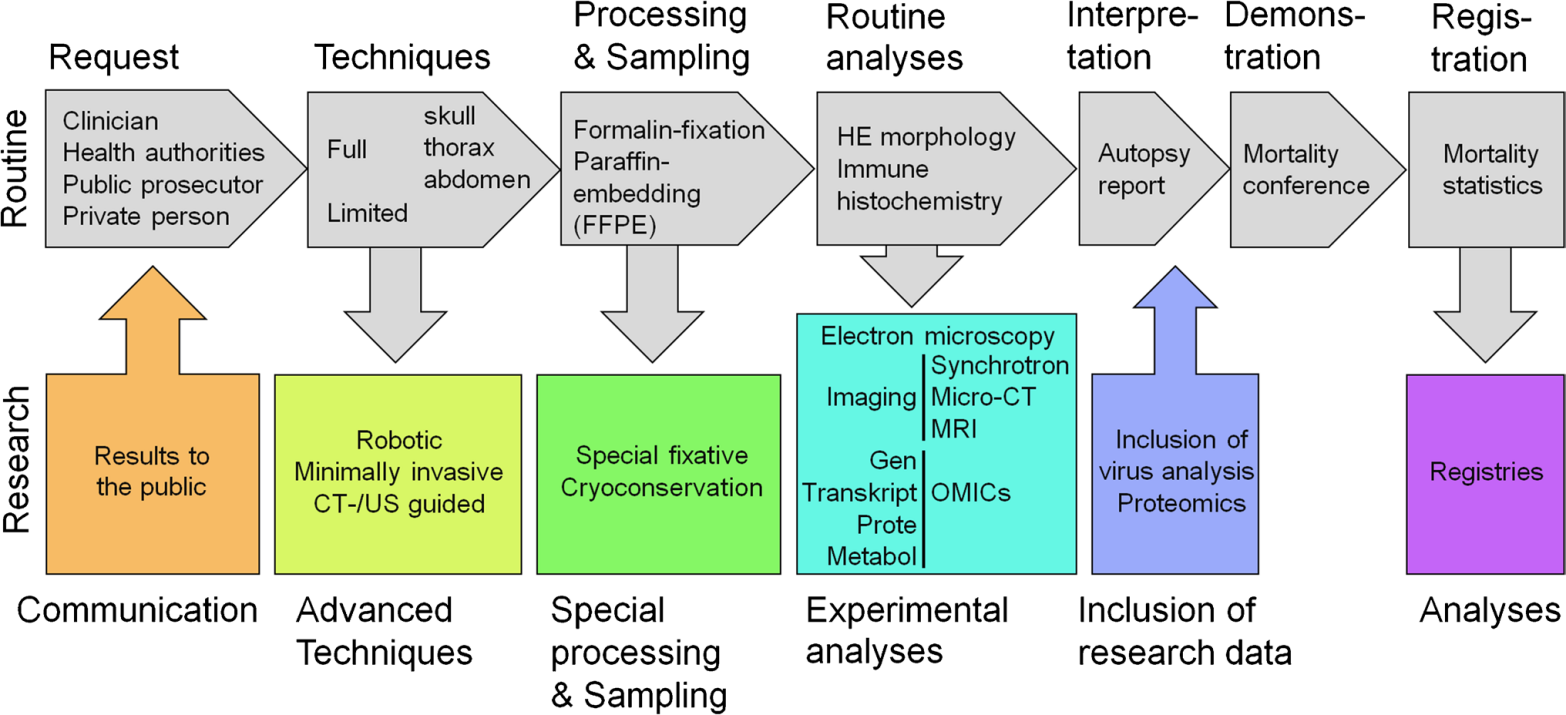
“Corpse journey” autopsy routine workflow and research applications in neuropathology, clinical pathology, and forensic pathology

The standard procedure is to undertake a complete autopsy with the opening and assessment of all three body cavities (cranium, thorax, and abdomen), followed by macroscopic and microscopic evaluation (Fig. 1). Alternative autopsy techniques are also being increasingly used, e.g., the so-called rapid autopsy, performed as soon as possible after death [13], or “minimal-invasive” autopsies, which use post-mortem imaging, e.g., computed tomography (CT)- or ultrasound to guide biopsy-based tissue sampling, which can be coupled with robotics [40, 86]. The advantages of these techniques include improved tissue quality and in part higher consent rates with relatives.

Standard biomaterial sampling during autopsy includes formalin-fixed and paraffin-embedded tissues, which can include whole organs, if necessary, such as the brain for neuropathological examination. Further biomaterials required for specific diagnostic purposes can include organ-specific swabs (performed in a sterile way), body fluids (e.g., venous blood, cerebrospinal fluid, urine), native and cryopreserved samples (e.g., for virus and bacteria isolation [20]), or samples for electron microscopy. In a forensic scenario, body fluids and samples are routinely retained for toxicological analyses. As a result, the autopsy examination provides unique access to human material from all organ systems, allowing an otherwise impossible comprehensive assessment of deceased patients. Specific sampling sites, fixation methods, and clinical data available in the German Registry for COVID-19 autopsies (DeRegCOVID), are outlined in detail in a retrospective nationwide multicenter registry study. This study analyzed the electronic data on COVID-19 autopsy cases available in the DeRegCOVID, which mainly included analysis of age and sex distribution, disease duration, postmortal SARS-CoV-2 detection and cause of death. The study also reports on experiences and other achievements of building-up such a registry [150]. In this review, 35 publications with a direct link to the network are included.

Several approaches can be used to detect infectious agents in (autopsy) tissues. The gold standard of SARS-CoV-2 diagnostics is the detection of its ribonucleic acid (RNA) using highly sensitive nucleic acid amplification techniques, mainly quantitative reverse transcription-polymerase chain reaction (RT-qPCR). Since the beginning of the pandemic, several (commercially) available tests have been validated and approved for use with patient swabs and respiratory secretions. These assays can also be adapted for analyses of (postmortem) tissue, either fixed or unfixed, to address challenges of RNA degradation [64, 129, 157]. Currently, however, no widely validated PCR tests for use in tissues are available. Compartment-specific testing proved useful in confirming suspected cases or understanding of viral spread in the human body [129, 160]. Immunohistochemistry and electron microscopy are challenging to interpret in autopsy-derived samples, cannot be recommended for routine nor research detection of SARS-CoV-2, and should be used and interpreted with caution. Specifically, establishment of the staining protocol including adequate positive and negative controls is crucial [89, 149]. In electron microscopy, a variety of cellular structures can easily mimic virus particles; the search for these can be highly time-consuming and the risk of false-positive interpretation is high. Therefore, it can only be recommended for research and diagnostics when applied and interpreted by experts in the field [68, 89].

With the appropriate preanalytical elements for tissue sampling, autopsy material can be analyzed using a broad armamentarium of modern technologies, allowing deeper insights into pathomechanisms and alternative approaches for virus detection alike. These include single cell or single nucleus RNA sequencing [35], in situ hybridization [69, 108, 129, 160], viral genome sequencing [138], gene-expression analysis on the RNA [8] and proteome level [117], innovative tissue imaging, and 3D reconstruction [8, 42] (Fig. 1).

**Fig. 2:**
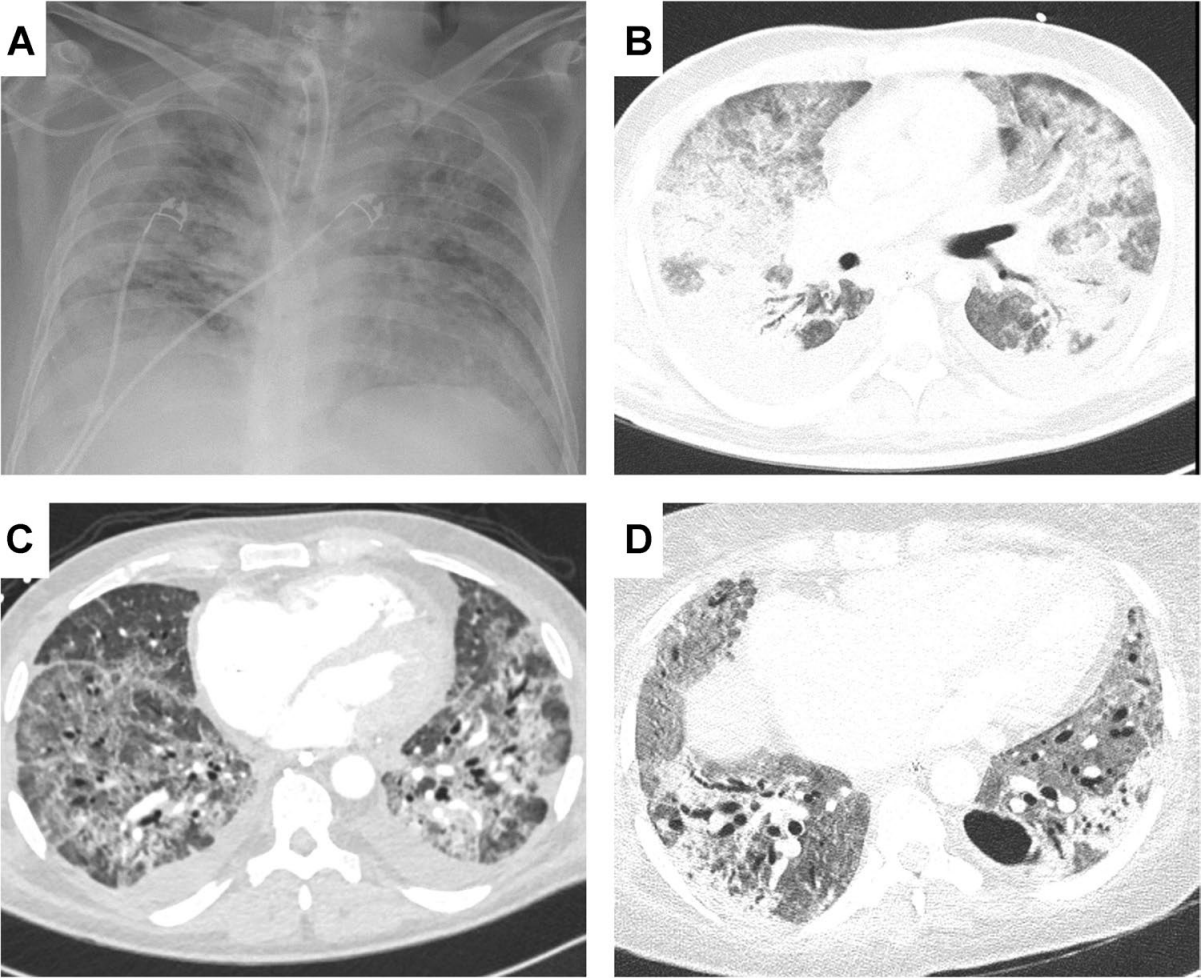
Radiographic images of pulmonary changes in COVID-19 over time: **A** Typical radiographic presentation of acute respiratory distress syndrome with bipulmonary infiltrates, so called “white lung”. **B** CT-image of a 39-year-old previously healthy man after 30 days of extracorporeal membrane oxygenation (ECMO) treatment displaying bipulmonary ground glass opacities and basal infiltrates in line with (prolonged) ARDS. **C** CT-image of a 62-year-old man showing interstitial fibrosis and cystic remodeling including subpleural areas after 3 weeks of ECMO treatment. **D** CT-image of 34-year-old woman after 70 days of ECMO treatment with advanced pulmonary remodeling

**Fig. 3:**
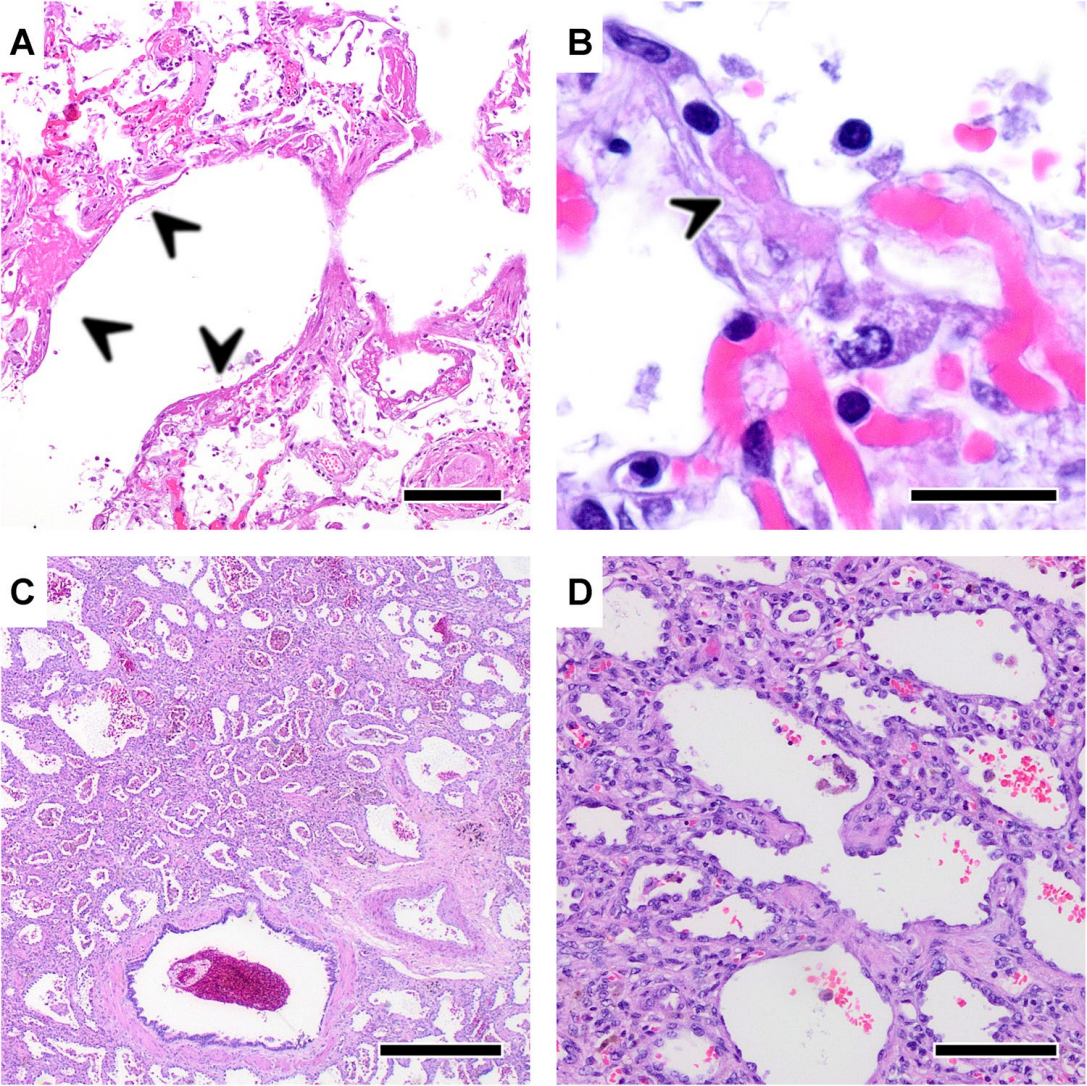
Histologic findings in acute (panel **A** and **B**) and post-acute (panel **C** and **D**) COVID-19. **A** Acute COVID-19 pneumonia with diffuse alveolar damage (DAD) characterized by hyaline membranes (arrows), alveolar septae necrosis and lymphocytic inflammatory infiltrate. HE staining, Magnification 50×, scale bar 200 μm. **B** Acute COVID-19 pneumonia with a capillary hyaline microthrombus (arrow). HE staining, Magnification 600×, scale bar 10 μm. **C**, **D** Post-acute COVID-19 fibrotic remodeling with thickened alveolar septae and prominent type-II-pneumocyte hyperplasia. HE staining, panel **C**: magnification 20×, scale bar 500 μm, panel **D**: magnification 100×, scale bar 100 μm

**Fig. 4:**
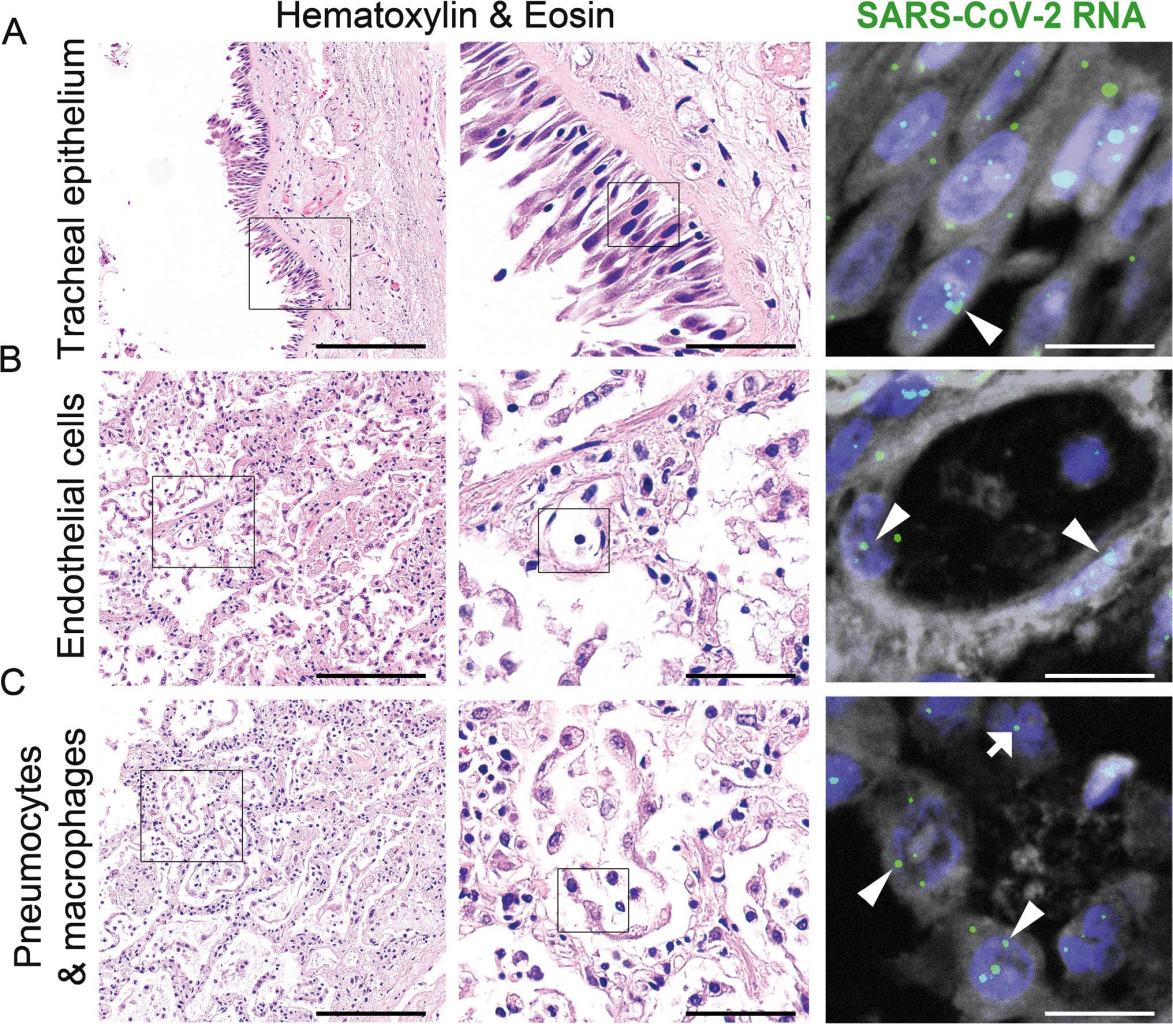
Respiratory tract morphology of COVID-19 patients with Hematoxylin & Eosin staining and SARS-CoV-2 RNA detection by fluorescence in situ hybridization, corresponding areas from consecutive slides. **A** Respiratory epithelial cells lining the tracheal mucosa with SARS-CoV-2 RNA (arrowhead, green signal). **B** Pulmonary alveolar capillary endothelial cells with detection of SARS-CoV-2 RNA (arrowheads, green signal). **C** Pulmonary intraalveolar detached pneumocytes (arrowheads, green signal) and intraalveolar macrophages (arrow, green signal) with SARS-CoV-2 RNA. Scale bars: left column, scale bars = 100 μm, center column, scale bars = 50 μm, right column, scale bars = 10 μm

In addition to a large body of data on molecular changes in tissues from COVID-19 patients, application of the above-mentioned techniques in autopsy tissues has also demonstrated protein expression that enable viral infection of the host cell, particularly the viral-entry receptor angiotensin-converting enzyme 2 (ACE2) and transmembrane protease, serine 2 (TMPRSS2), the cell-membrane-based protease needed for conformational changes in the SARS-CoV-2 spike protein leading to fusion with the host cell plasma membrane [152]. These proteins were found in most human cell types and most tissues, including epithelial, endothelial, mesenchymal, immune, muscular, and neural cells [60, 67, 160, 175]. Notably, there are tissue and cell-specific differences regarding ACE2 and TMPRSS2 expression that further vary upon underlying diseases. Cardiomyocytes, for example, express significant levels of the ACE2 receptor [173] but lack TMPRSS2 [166], thus, questioning direct cardiomyocyte infection. Endothelial cells, on the other hand, do express both receptors and thus might facilitate cardiac involvement in COVID-19. However, ACE2 expression in a normal liver is minimal and mainly restricted to bile ducts, but is significantly upregulated in hepatocytes in liver fibrosis, hinting at a higher prevalence of liver involvement in cirrhotic patients [119]. In addition, virus factors also play a role in organ tropism and disease involvement. The currently dominant Omicron variant has a higher affinity for ACE2 compared to Delta and inefficiently utilizes the cellular protease TMPRSS2 that promotes cell entry via plasma membrane fusion; thus, it is dependent on cell entry via the so-called endocytic pathway. These alterations might ultimately change the tropism from ACE2/TMPRSS2 co-expressing cells to ACE2-only expressing cells resulting in a different clinical presentation and organ involvement [109]. These findings underline the crucial role of ongoing autoptic investigations in the ongoing pandemic (Figs. 2, 3, and 4).

## Organ specific involvement in COVID-19

### Lung and respiratory system

COVID-19 is associated with a broad spectrum of clinical respiratory syndromes ranging from mild upper airway symptoms, to progressive viral pneumonia leading to labored breathing and progressive hypoxemia. Intensive care treatment with invasive mechanical ventilatory support is required in approximately 10% of hospitalized cases [39, 76]. Pulmonary manifestation is the leading cause of death in COVID-19 patients, usually presenting with peripheral lung ground-glass opacities on computed tomography (CT) [131, 145], similarly detectable with post-mortem imaging in autopsies [86]. Histologically, this corresponds to the morphological hallmark of acute respiratory distress syndrome (ARDS), i.e., diffuse alveolar damage (DAD) with edema, hemorrhage, hyaline membranes, and pneumocyte damage [77].

The diffuse alveolar damage in SARS-CoV-2 [18, 135], and similarly in SARS-CoV [15, 101, 115], is associated with a prominent formation of fibrin thrombi. SARS-CoV-2-infection leads to an angiocentric inflammation in COVID-19-induced respiratory failure with a greater number of ACE2-positive endothelial cells [8]. Increasing clinical evidence shows that endothelial dysfunction is a common denominator after SARS-COV-2 infection [71]. Histologically, this is characterized by acute vascular inflammation and perivascular T cell recruitment leading to swelling and disruption of the endothelial cell barriers and an anomalous microvascular architecture [8, 71]. The resulting vascular injury, especially of endothelial cells [147] leads to (micro-) thrombosis [94], vasoconstriction, and intussusceptive angiogenesis. Intussusceptive, i.e., non-sprouting, angiogenesis is a morphogenetic process of intravascular septation that produces two lumina from a single vessel within minutes, and has so far been identified in cancer [5], inflammatory and autoimmune diseases and tissue regeneration [3]. Intussusceptive angiogenesis was increased threefold in COVID-19 patients compared to influenza A virus subtype H1N1 (A/H1N1, “swine flu”) associated ARDS and manifested early (within days) but persisted over 20 days after initial infection.

Pulmonary thrombosis in large vessels was only seen in some of the cases, whereas most showed obstructed microvasculature by fibrin strands, activated platelets, deformed neutrophils, and neutrophil extracellular traps [95, 125]. Although virus-associated thrombotic microangiopathies have been described in several cardio-respiratory diseases, e.g., influenza [142] or Parvo-B19-viral myocarditis [9], in COVID-19 lungs these pathologies were reported up to nine times more often compared to influenza A/H1N1 lungs [8]. The extensive microangiopathy observed in COVID-19 patients leads to hypoxia, intrapulmonary shunting via upregulation of the bronchial vasa privata [42], and an overall increase of pulmonary vascular resistance [125]. These autopsy findings were a turning point in the understanding of COVID-19 as an angiocentric disease providing potential explanation for the drastic and rapid clinical deterioration of COVID-19 patients. The massive impact on the microvasculature also provided an explanation for the difficulties observed in the management of mechanical ventilation and the relatively high need for extracorporeal membrane oxygenation therapy [14]. These histology-based autopsy studies were the propelling factor for the adaptation of anti-thrombotic treatment in COVID-19. The above-mentioned structural adaptation of the microvascular network, transmigration of lymphocytes, a shift toward M2 macrophages [170], and the “cytokine storm” observed in COVID-19 patients is considered as a response to SARS-CoV-2-induced cellular damage. This is supported by multimodular detection of viral components in endothelial cells [4, 8], lymphatic cells [104], but also, in type 1 and 2 pneumocytes [19, 104]. Mucus plugging was noted at autopsy in COVID-19 patients with and without history of asthma [88, 110].

Prominent pulmonary microvascular alterations observed in COVID-19 resemble those of distinct fibrotic lung diseases [6, 41, 91]. These findings are in line with histologic changes reported through the course of disease, from acute DAD to organizing changes with interstitial myofibroblastic proliferations, septal collagen deposition, and development of loose alveolar plugs of fibroblastic tissue [17]. Although the pathologic mechanisms underlying fibrotic remodeling in pulmonary thrombotic or thromboembolic occlusions are still not fully understood, thrombo-fibrosis appears to be promoted by hypoxia-induced activation of endothelial cells, incorporation of mesenchymal progenitor and immune cells with subsequent scarring of the pulmonary parenchyma [2, 6, 7, 164].

As the nasopharyngeal and oropharyngeal tissues are the main entry and (early) replication sites of SARS-CoV-2 [159], it was shown that SARS-CoV-2 infects respiratory, olfactory, and paranasal sinus epithelia with tropism to ciliated mucosa [112], and the adjacent tissues, and can explain the reason why anosmia and dysgeusia are prevalent symptoms in infected patients [85]. A similar pathogenesis has been proposed in the salivary gland, in which SARS-CoV-2-infected glands show inflammation, injury of the glandular parenchyma, and abnormalities in the saliva [100, 140]. Other hypotheses proposed the vagus nerve as a virus pathway from the respiratory to the central nervous system [163].

### Cardiovascular system

Acute cardiac involvement, i.e., elevated cardiac injury markers such as troponin, arrhythmia, lowered ejection fraction, ventricular dyskinesia, etc., has been variably reported from 16% [154] to 36% [78] of COVID-19 patients on clinical admission and up to 78% in one magnetic resonance imaging (MRI) cohort-study [130]. These frequencies are in line with those reported for severe lung disease caused by the closely related betacoronavirus SARS-CoV [167].

At the beginning of the COVID-19 pandemic—especially in children—a vasculitis-like symptom complex with occasional cardiac involvement, partly resembling Kawasaki disease, was reported and termed (pediatric) inflammatory multisystem syndrome (P)IMS [31, 53], however, so far, the concept of (P)IMS is still debated.

Current hypotheses on cardiac involvement include direct injury to the myocardium by a viral infection, given that cardiomyocytes express significant levels of ACE-2 [173]. However, cardiomyocytes lack TMPRSS2 [166], questioning relevant direct cardiomyocyte infection. Another explanation for the cardiac involvement is the systemic release of pro-inflammatory mediators, e.g., interleukins (IL-1, IL-6), tumor necrosis factor alpha (TNF-α), interferon-γ (IFN-γ), and macrophage inflammatory protein (MIP), often called the cytokine storm [27, 36]. The mechanisms of indirect myocardial injury include increased vascular wall permeability and myocardial edema, which are in line with observed wall thickening in ultrasound or MRI, and a patchy hypoxia-induced inflammatory response and cellular damage [168]. Evidence for a direct SARS-CoV-2-specific cardiac involvement is based on a series of case reports describing clinical (peri-) myocarditis [65, 70, 73, 82, 168]. In the majority of these studies, except two reports on lymphocytic myocarditis [37, 51], the histopathological assessment did not show typical signs of viral myocarditis according to the Dallas criteria, i.e., lymphocyte infiltration and myocyte necrosis. Instead, increased numbers of macrophages were observed, often termed as “borderline myocarditis” [11, 59, 144, 155]. The presence of SARS-CoV-2 RNA in cardiac tissue has been reported, but the extent and compartment specific involvement are still debated [11, 51, 144, 157]. In analogy to overt vascular involvement in the lungs, cardiac endothelialitis was proposed either via direct virus infection [49, 50, 144] or indirect mechanisms [16, 58, 122]. An autopsy study of 95 COVID-19 cases failed to demonstrate myocarditis, but showed relevant SARS-CoV-2 virus loads in 43% of all cases, where the virus was primarily located in interstitial cells. Cardiac virus replication was found in 15% of these cases [96]. On the other hand, SARS-CoV-2, particularly the Omicron variant, seems to be able to enter cells independently of TMPRSS2 by the so-called endocytic pathway [109]. There are single case reports of virus-shaped particles [11] in cardiomyocytes; thus, direct infection might be possible in a subset of cases. However, the clinicopathological evidence remains scarce [24]. Another finding in COVID-19 autopsies was cardiac amyloidosis. Cardiac amyloidosis is not uncommon in autopsies, particularly in elderly patients, and is often not associated with clinical symptoms [124]. A direct pathophysiological link between COVID-19 and cardiac amyloidosis is debated, but appears to be rather unlikely. However, cardiac amyloidosis might predispose COVID-19 patients to heart failure regarding the higher incidence of amyloidosis seen in this cohort [62].

Altogether, it is not yet clear whether cardiac involvement in COVID-19 follows the classical inflammatory pathogenesis observed in myocarditis caused by other viruses, such as influenza virus, SARS-CoV-1, or coxsackie virus or whether microvascular/angiogenic or inflammatory pathogenesis might rather be the driving forces in COVID-19 cardiac involvement.

### Kidneys

Kidney involvement is among the most frequent and severe organ complications in severe COVID-19, mostly manifesting as acute kidney damage and being associated with unfavorable outcome [20]. Next to diabetes, cardiovascular disease, and chronic respiratory disease, chronic kidney disease is the most important underlying disease associated with risk of severe COVID-19 [30]. Besides, patients with chronic kidney disease, dialysis, or kidney transplant are particularly vulnerable to COVID-19 with high morbidity and mortality [74]. Renal SARS-CoV-2 tropism was first demonstrated in autopsies [129] and is associated with increased disease severity and acute kidney injury [20]. Affected kidneys in most cases show acute tubular damage, followed by collapsing glomerulopathy or, likely unspecific or secondary, focal segmental glomerulosclerosis [48]. Microthrombi or thrombotic microangiopathy were observed but are difficult to attribute directly to the SARS-CoV-2 infection [146]. Specific proximal tubular dysfunction has also been described [156]. So far, it is unclear to what extent the renal involvement is mediated via direct viral effects on kidney cells, secondary effects of a severe course of disease with cytokine storm and hypoxia, or a combination thereof.

### Immune, lymphatic, and hematopoietic system

The inflammatory reaction in COVID-19 shows some differences to other viral lung diseases, such as influenza. The initial inflammatory response occurs earlier in patients infected by influenza while the intensity of inflammation is generally comparable. In contrast to influenza, one hallmark of COVID-19 is the missing or delayed limitation of the immune response due to prolonged viral persistence or impaired endothelial function and microcirculation resulting in increased risk of secondary complications and fibrotic remodeling [23]. The impaired immune response in severe COVID-19 seems to be the result of immature, dysfunctional neutrophils, and monocytes [136]. The high proportion of asymptomatic or mild disease courses in COVID-19 and the rapid immune response and production of antibodies following vaccination may be explained by cross-reactive immunity with other coronavirus strains [21, 99].

A common phenomenon in lymphatic tissues following viral infections is the expansion of the paracortical areas with extrafollicular activation of B cells caused by antigen-specific activated T cells. This extrafollicular B cell activation is the morphological correlate of rapid B cell responses and is accompanied by rather small, inactive germinal centers [22, 63]. Upon recognition of an antigen, B cells differentiate into short-lived antibody-forming cells, are active as frontline protectors, do not participate in class-switch, and are not able to differentiate to memory B cells [22, 26]. Lymph nodes of COVID-19 patients exhibit these features and are characterized by extrafollicular accumulation of plasmablasts [158, 171] and a decrease of memory B cells [61, 114].

Peripheral lymphopenia with loss of memory B cells is a typical feature of severe COVID-19, partly due to a redistribution of lymphocytes from lymphatic organs to the lung [33, 54, 102]. Lymphatic depletion may also be seen in the lymph nodes and the spleen due to a direct cytopathic effect of the virus [1]. In these patients, the absence of germinal center reaction is accompanied by a striking reduction in B cell lymphoma 6 protein (BCL6) positive germinal center B cells and also an early specific block in BCL6-positive T helper cell differentiation, together with an increase in cytokine-producing T_H1_ cells and aberrant extrafollicular TNF-α accumulation [75]. Also, a dominant population of CD8-positive T cells and a diffuse increase in M2 polarized macrophages have been described [61, 118].

Patients with a severe COVID-19 course exhibit a systemic inflammatory response with an altered pattern of inflammatory chemokines, cytokines, high ferritin levels, and IL-1/IL-6 pathway dysregulation. Although these features are not specific for COVID-19 [47, 98, 126], cytokine dysregulation plays an important role in the progression of severe COVID-19. In the context of the cytokine storm, hemophagocytosis in lymph nodes, spleen, and bone marrow have been put forward as possible histomorphologic features, although it is not observed universally [97, 174].

Secondary hemophagocytic lymphohistiocytosis, cytokine release syndrome, and macrophage activation syndrome are overlapping syndromes characterized by an activation of lymphocytes and macrophages with a subsequent excessive immune response, which leads to multiorgan damage. In addition, most reports describe an increase of bone marrow cellularity with left shift deviation. The increase in the total numbers of macrophages might be due to the prominent systemic inflammation, while the increase in CD8^+^ T cells is considered a direct effect of viral infection [38, 57, 107, 127]. However, histiocytic hyperplasia with secondary hemophagocytic lymphohistiocytosis was primarily seen in autopsy studies indicating terminal dysfunction, but not in those patients surviving the infection showing decreasing IL-6 levels [118, 126].

The concept of neutrophil extracellular trap formation (NETosis), followed by activation of the coagulation cascade, a process termed “immunothrombosis,” has been recognized as a pathogen eradication strategy that could play a central role inducing or exacerbating autoimmune or vascular pathologies in COVID-19 and other inflammatory conditions [116, 177].

### Liver, pancreaticobiliary, and gastrointestinal system

Morphological alterations of the liver are quite common in living and deceased COVID-19 patients; however, they are mostly unspecific. Steatosis, mostly macrovesicular, is frequent and with a prevalence of 30–70% likely represents a preexistent finding and/or a possible result of hypoxia and drug-related adverse effects rather than a virus-induced pathology [46, 113]. Other changes, such as mild lobular hepatitis, centrilobular congestion and necrosis, platelet-fibrin microthrombi (15–70% of the cases), and cholestasis are likely related to the cytokine storm, the general hypoxia in COVID-19 patients, and septic complications [44, 46, 172]. Although in some studies viral RNA could be detected in a substantial number of liver samples [92], this result could not be confirmed by others [105].

Pancreatic enzyme elevation and acute pancreatitis have been described in SARS-CoV-2-infected patients, especially in severe and critical manifestations of COVID-19 [176]. While a potential causal relationship between viral infection and pancreaticobiliary organ damage is still not fully understood [32], it was shown that SARS-CoV-2 can infect and replicate in ex vivo in cultured human pancreatic islets, which was associated with morphological, transcriptional and functional changes [111, 161]. In autopsy studies, severe injury or remodeling of the pancreas during COVID-19 has not been reported so far [44]. Detailed examination, especially of the pancreas, is often hampered by its characteristic rapid post-mortem autolysis. A recently described severe manifestation of cholangiopathy, so severe that it might result in liver transplantation, most likely represents a variant of secondary sclerosing cholangitis in critically ill patients [45, 84].

Gastrointestinal symptoms such as anorexia, diarrhea, nausea, and vomiting have been reported in about 15% of patients with COVID-19 and some cases presenting with isolated gastrointestinal manifestation have been observed [56]. Viral RNA can be detected in fecal samples from patients with COVID-19, and viral RNA positivity can persist even after sputum samples become PCR negative [153]. Although fecal transmission was possible in a laboratory system as shown by infection of colon carcinoma cell lines (CACo) and gut organoids [93]. The true risk and relevance of SARS-CoV-2 transmission via a fecal-oral route remains unclear and seems to be of rather limited importance. Immunohistochemistry and single-cell transcriptome data revealed expression of ACE2 and TMPRSS2 in enterocytes of the small intestine and colon, most abundant in the ileum [60, 169]. Data of endoscopic samples or surgical specimens is sparse and so far, consists only of small case series or case reports. In COVID-19 patients with gastrointestinal symptoms, structural damage has been variable and ranges from limited and focal inflammation with interstitial edema accompanied by plasmacellular and lymphocytic infiltrates, up to substantial ulceration and necrosis of the mucosa. Notably, different studies confirmed viral RNA and antigens in intestinal epithelial cells as well as in macrophages and lymphocytes suggesting active SARS-CoV-2 replication in the intestine.

### Nervous system and skeletal muscle

SARS-CoV-2 invasion in the central nervous system (CNS) occurs via the blood or nerves. A hematogenous route is supported by the fact that COVID-19 leads to viremia and SARS-CoV-2 targets brain endothelial cells [106, 108, 139]. Also, SARS-CoV-2 is not only transported through brain endothelial cells but also replicates in these [90]. The olfactory route and transport along vagal nerves have been suggested as a CNS entry port. Dysfunction of the olfactory system is a key symptom of COVID-19 and SARS-CoV-2 colonizes the nasal cavity. If SARS-CoV-2 additionally transits to CNS via olfactory and sensory nerve endings in the olfactory mucosa is debated with data in favor and opposing this concept [80, 108]. In COVID-19, there is vagal nerve dysfunction and SARS-CoV-2 viral proteins can be found in COVID-19 patients, thus a vagal route of SARS-CoV-2 CNS entry has to be considered [25, 52].

When considering the larger neuropathological studies [34, 106, 108, 132, 139], focal cerebral infarctions are seen in approximately 13% of autopsies. However, cerebral hypoxia in COVID-19 is not consistently defined so far. Global hypoxic-ischemic states, possibly resulting from respiratory failure in COVID-19, have to be distinguished from focal cerebral thromboembolic events and patients with systemic hypercoagulation syndrome. The cytokine storm results in a deterioration of the blood-brain barrier. In contrast to the microglial activation reported in many studies, only a few cases with encephalitis or meningitis have been described [29]. Microglia and astrocyte subpopulations associated with COVID-19 share features with those found in neurodegenerative diseases [137, 165], and synaptic signaling of upper-layer excitatory neurons, linked to cognitive function, are preferentially affected in COVID-19 [165]. Since patients with COVID-19 frequently succumb to bacterial superinfection, sepsis, ventilation, and polypharmacotherapy as well as long-term intensive care unit (ICU) stays with an isolating environment/deafferentation, comparative studies with similar clinical pictures will be required to identify COVID-19 specific encephalopathic changes. Up to now, there is no evidence of a CNS reservoir for viable SARS-CoV-2 virus [90], even though viral RNA or protein may be found at the CNS barriers. A future challenge is the identification of how the CNS contributes to symptoms of the post-COVID syndrome, such as fatigue, headaches, anosmia, muscle weakness, and cognitive dysfunction; it is still very early days. Although there are already large studies on post-COVID, with thousands of study participants [143], the time span is not sufficient to be able to say how long-term consequences will develop. The patients presenting with neurological signs of post-COVID are probably a heterogenous group with some having a dysregulated microbiome, others alteration of the vascular system or dysfunctional brainstem signaling, as well as others with ongoing low-level inflammation or autoimmunity triggered in susceptible hosts [128].

COVID-19 can affect the peripheral neural system meeting diagnostic criteria for acute polyradiculoneuropathy. Early in the course of the pandemic, it was suggested that similar to other viruses, SARS-CoV-2 might directly infect peripheral neurons or trigger Guillain-Barré syndrome (GBS) [55, 141]. However, later epidemiological studies found no link of GBS and SARS-CoV-2 [79]. Similarly, direct infection of skeletal muscle fibers or autoimmune myopathy/myositis triggered by SARS-CoV-2 have been suggested to cause myalgia, muscle weakness, and elevated creatine kinase (CK) levels that are frequently observed in COVID-19 patients [55] and were more pronounced in critically ill patients, compared to mildly affected individuals [103]. A post-mortem case control study could not detect signs of infection of skeletal muscle, but identified myositis with different levels of severity in COVID-19. Inflammation of the muscle was correlating with disease duration, supporting a postinfectious, immune-mediated pathology [12].

## Endocrine organs

Thyroid dysfunction in COVID-19 occurs frequently, ranging from thyrotoxicosis, observed in 15 to 20% of patients, to hypothyroidism and nonthyroidal illness syndrome (NTIS) [28, 81]. The causes of thyroid impairment remain elusive and a multitude of putative explanations has been considered. Direct infection of thyroid follicular epithelium appears as a putative pathogenic process, given the high ACE2 expression compared, e.g., to the lungs [134]. However, studies analyzing SARS-COV-2 RNA or protein yielded contradictory results, with some studies reporting the presence of viral components [160], while others do not [28, 134]. Histologic analyses of tissues obtained from autopsies yielded conflicting results and the thyroid, as well as other endocrine organs, have not yet been sufficiently investigated in most autopsy studies [121, 134]. The presence of interstitial lymphoid infiltrates in the thyroid, commonly reported in COVID-19, is difficult to differentiate from pre-existing thyroiditis and is thus of unclear significance [134]. Regardless of the underlying pathogenesis, the degree of thyroid dysfunction directly correlates with the patients’ prognosis. The current knowledge concerning the effect of SARS-CoV-2 on other endocrine organs, such as the pituitary gland, parathyroid, adrenals, gonads, endocrine pancreas, and the diffuse neuroendocrine system, remains rudimentary [121].

## Superinfections

SARS-CoV-2 induces epithelial damage, leading to impaired epithelial barrier function and consecutive invasion by secondary pathogens [123]. In a clinical context, invasive ventilation and other complications (e.g., sepsis and multi organ failure) can further aggravate those alterations. Overall, however, superinfections are not increased in the early phase of COVID-19 disease, occur with similar frequency as in other systemic viral infections (e.g., influenza), and are usually acquired via the respiratory system [72]. The combination of downregulation of proteins playing a key role for the innate immune system, e.g., the toll-like receptors, and corticosteroid treatment in severe COVID-19, might contribute to the increased susceptibility for life-threatening mycosis and bacterial superinfections with sepsis, especially in patients with long-term intensive care treatment for SARS-CoV-2 infection [10, 44, 61, 87, 120].

## Open questions, limitations, and conclusions

Since the start of pandemics, our understanding of COVID-19 has greatly increased. Besides tremendous efforts in clinical and virological research, data derived from clinical and forensic autopsies represent an important pillar of the rapid increase in knowledge. Most autopsy-based findings provide information on the severe and later stages of the disease and help to delineate discrepancies between clinical presentation and actual organ involvement, acting as key drivers for the development of novel diagnostic and therapeutic tools, as well as feedback on the individual patient.

The value and importance of autopsies increased tremendously during the COVID-19 pandemic. This rejuvenated the field of clinical autopsies, which was slowly becoming an ever-diminishing branch of pathology. It also led to a substantial increase in recognition of the broad applicability of most novel technologies of biomedical research, even in post-mortem and autopsy tissues, resulting in data going far beyond the classical histopathological analyses. The use of these methods predicts a very clear-cut future for the further development of autopsies beyond COVID-19. However, it also became apparent that for many biomaterials and data, we are currently lacking comparable or matched control or reference autopsy cohorts. An increment of clinical autopsies is a mandatory stepping-stone as preparation for the next pandemic—and for the interim period—to gather reference values and biomaterials and enable optimal training of surgical and forensic pathologists.

The current immense knowledge on COVID-19 was only possible via interdisciplinary, systematic, and holistic analysis of findings from clinical observations, imaging, laboratory diagnostics, and autopsies. However, there is a considerable risk of over-interpretation of (unspecific) findings. So far, no other disease has been studied as extensively in such a short period of time and with such intensity by dozens of specialists. It is not surprising that many of the current studies, not only on autopsies, have limitations that have to be kept in mind. These include single-center designs, often with limited numbers of cases, and, importantly, lacking control populations. Bringing together national and international collaborative initiatives seems inevitable to effectively tackle the COVID-19 pandemic and serve as a preparedness infrastructure for the future. The German initiatives, the Network for Autopsies in Pandemics (DEFEAT PANDEMIcs) and the COVID-19 Autopsy Registry (DeRegCOVID), are national examples, demonstrating that joint approaches are feasible, effective, and can serve as a blueprint for other countries. These examples should serve as an initiator for international initiatives, which are being conducted in clinical medicine, such as the Solidarity study of the WHO (World Health Organization), but not yet in pathology. In addition, the network operates in an integrative, participatory, and interdisciplinary manner including virologists, biologists, and clinicians and is open to all new collaborations and complementary expertise.

Important open questions for future research include the (long-term) consequences of COVID-19, especially post-COVID conditions (“long COVID”). Autopsies and molecular methods will likely help to unravel the mechanisms of these conditions in specific organ systems. This might include viral persistence in tissues, aberrant immune phenotypes of resident cells, or even auto-immune processes. Additional open questions also include the (immune) pathological role of virus variants. The DEFEAT PANDEMIcs consortium recently started a detailed post-mortem virus sequencing in fatal cases. First autopsy reports indicate no differences in virus loads and organotropism between the wild type and the first relevant virus variant, B.1.1.7 [64].

In conclusion, the direct pulmonary effects of SARS-CoV-2 infection are well established and are the leading pathology in COVID-19, with data indicating disease specific mechanisms such as angiocentric inflammation with systemic (micro-) thrombosis and neoangiogenesis via intussusception. In comparison, it remains unclear to what extent virus invasion or replication might contribute to extrapulmonary organ injury, e.g., in the kidney. Such pathologies, including the observed hypercoagulability, might represent indirect effects of severe disease course and multi-organ failure, or the combination of both direct and indirect effects. Future joint studies integrating the knowledge and material acquired by autopsies, together with clinical studies, preclinical models, and humanized organoid systems, are needed to dissect these emerging questions.

## Supplementary Information

Below is the link to the electronic supplementary material.

Supplementary Table 1 COVID-19 Autopsies in DEFEAT PANDEMIcs consortium (DOCX 16 KB)
