# Supplementary material for: Organ manifestations of COVID-19: what have we learned so far (not only) from autopsies?
Source: Virchows Arch. 2022 Apr 1;481(2):139–59. doi: 10.1007/s00428-022-03319-2 (PMC8975445; doi:10.1007/s00428-022-03319-2)
Supplement: Supplementary file 1 — COVID-19 Autopsies in DEFEAT PANDEMIcs consortium (DOCX 16 KB) [file 428_2022_3319_MOESM1_ESM.docx]

Supplementary Table 1: COVID-19 Autopsies in DEFEAT PANDEMIcs consortium

|  | Type of autopsy | | | | Patient characteristics | | Cause of Death |  |
| --- | --- | --- | --- | --- | --- | --- | --- | --- |
| Center | Clinical (n) | Officially ordered (n) | Forensic (n) | Insurance (n) | Male (n)/ female (n) | Age (y;min - max, mean) | COVID-19 Cause of Death (%) | COVID-19 Cause of Death (N) |
| Aachen | 22 | 0 | 0 | 1 | 16/7 | 67 (43-92) | 87 | 20 |
| Augsburg | 140 | 2 | 0 | 0 | 53/89 | 75 (18 - 97) | 90 | 128 |
| Berlin | 106 | 0 | 15 | 1 | 90/32 | 69 (21-97) | 82 | 100 |
| Bielefeld | 28 | 0 | 0 | 0 | 10/18 | 82 (61-97) | 75 | 21 |
| Düsseldorf | 12 | 1 | 10 | 2 | 16/9 | 69,56 (34-94) | 76 | 19 |
| Dresden | 48 | 2 | 0 | 0 | 35/15 | 76 (33-92) | 88 | 44 |
| Erlangen | 24 | 0 | 0 | 1 | 16/9 | 72 (26-96) | 80 | 20 |
| Frankfurt | 2 | 0 | 29 | 0 | 18/13 | 74,3 (38-96) | Pending | Pending |
| Freiburg | 16 | 0 | 0 | 0 | 9/7 | 63 (43-77) | pending | Pending |
| Giessen | 0 | 4 | 8 | 0 | 6/6 | 79 (59-99) | pending | Pending |
| Göttingen | 16 | 0 | 0 | 0 | 11/5 | 70 (39-98) | 75 | 12 |
| Halle | 15 | 0 | 0 | 0 | 10/5 | 65,5 (36-85) | 66,67( in 33,33 cases still unclear) | 10 |
| Hamburg | 9 | 137 | 45 | 58 | 137/112 | 77 (21-99) | 91 | 227 |
| Hannover | 9 | 0 | 0 | 0 | 9/0 | 64 (41-91) | 66 | 6 |
| Heidelberg | 69 | 9 | 0 | 3 | 58/23 | 72.8 (41-95) | 77 (58/75); (6 pending) | 58 |
| Homburg (Saarland) | 18 | 0 | 7 | 0 | 17/8 | 76 (55-96) | 60 | 15 |
| Rostock | 31 | 10 | 3 |  | 26/18 | 71 (23 - 91 ) | 74 - 86 (in 5 cases still unclear) | Pending |
| Jena | 60 | 3 | 11 | 0 | 48/26 | 71,3 (19-91) | > 80 | 59 |
| Köln & Leverkusen | 19 | 0 | 7 | 0 | 17 / 9 | 56 - 86 (mean 73) | 61 | 16 |
| Kiel | 71 | 2 | 0 | 7 | 52/28 | 75 (51-98) | 90 | 72 |
| München (LMU) | 36 | 0 | 0 | 0 | 27/9 | 70 (50-92) | 94 | 34 |
| München (TU) | 22 | 1 | 0 | 0 | 18/5 | 69 (38-92) | 91 | 21 |
| Münster | 9 | n.a. | n.a. | n.a. | 5/4 | 70 (61 - 87) | 78 | 7 |
| Mainz | 2 | 1 | 5 | 0 | 5/3 | 62 (37-81) | 75 | 6 |
| Tübingen | 26 | 1 | 0 | 0 | 20/7 | 63 | 81 | 22 |
| Regensburg | 27 | 0 | 0 | 0 | 17/10 | 67 (44 - 84) | 74 - 96 (6 cases still unclear) | Pending |
| Würzburg | 19 |  | 0 | 0 | 11/8 | 61 (33-97) | 89 | 17 |
| Sum | 856 | 173 | 140 | 73 |  |  |  | 933 |
| Total | 1242 |  |  |  |  |  |  |  |
